# The emergence of semantic categorization in early visual processing: ERP indices of animal vs. artifact recognition

**DOI:** 10.1186/1471-2202-8-24

**Published:** 2007-04-05

**Authors:** Alice M Proverbio, Marzia Del Zotto, Alberto Zani

**Affiliations:** 1 Department of Psychology, University Milano-Bicocca, Milan, Italy; 2 Institute of Molecular Bioimaging and Physiology, CNR, Milan-Segrate, Italy

## Abstract

**Background:**

Neuroimaging and neuropsychological literature show functional dissociations in brain activity during processing of stimuli belonging to different semantic categories (e.g., animals, tools, faces, places), but little information is available about the time course of object perceptual categorization. The aim of the study was to provide information about the timing of processing stimuli from different semantic domains, without using verbal or naming paradigms, in order to observe the emergence of non-linguistic conceptual knowledge in the ventral stream visual pathway. Event related potentials (ERPs) were recorded in 18 healthy right-handed individuals as they performed a perceptual categorization task on 672 pairs of images of animals and man-made objects (i.e., artifacts).

**Results:**

Behavioral responses to animal stimuli were ~50 ms faster and more accurate than those to artifacts. At early processing stages (120–180 ms) the right occipital-temporal cortex was more activated in response to animals than to artifacts as indexed by posterior N1 response, while frontal/central N1 (130–160) showed the opposite pattern. In the next processing stage (200–260) the response was stronger to artifacts and usable items at anterior temporal sites. The P300 component was smaller, and the central/parietal N400 component was larger to artifacts than to animals.

**Conclusion:**

The effect of animal and artifact categorization emerged at ~150 ms over the right occipital-temporal area as a stronger response of the ventral stream to animate, homomorphic, entities with faces and legs. The larger frontal/central N1 and the subsequent temporal activation for inanimate objects might reflect the prevalence of a functional rather than perceptual representation of manipulable tools compared to animals. Late ERP effects might reflect semantic integration and cognitive updating processes. Overall, the data are compatible with a modality-specific semantic memory account, in which sensory and action-related semantic features are represented in modality-specific brain areas.

## Background

Dissociation of neural activity related to processing objects belonging to different semantic categories has been demonstrated in several neuropsychological and functional neuroimaging studies [for review see [1-3]]. For example, Tranel et al. [4] demonstrated that word retrieval in response to visually presented concrete entities engages neural systems in the left temporal lobe and that the precise pattern of activation in the temporal lobe depends in part on the conceptual category to which the entity belongs. For instance, they found that patients with defective retrieval of concepts for persons had brain lesions that extended to the right temporal polar region, patients with defective retrieval of concepts for animals frequently had lesions in right mesial occipital and mesial ventral temporal regions, and patients with abnormal retrieval of concepts for tools had lesions that were localized to the left lateral occipital-temporal-parietal junction. According to the authors, their findings support the hypothesis that normal retrieval of semantic information about concrete entities from different conceptual domains depends on partially segregated neural systems.

Warrington and colleagues[5,6] proposed a model of representation for conceptual knowledge called *modality-specific semantic memory *that explains why damage to a particular modality-specific semantic system results in a specific impairment for a given semantic category, such as animals or persons. According to the model, dissociation of different semantic domains occurs because of the different types of perceptual and sensorimotor information that are associated with entities of different semantic categories. Thus, the representation of artifacts is more strongly associated with functional and action-related features, whereas the representation of natural objects is more strongly associated with sensory and perceptual features. The results of several functional neuroimaging and electrophysiological studies support the *modality-specific semantic memory *model [7-14]. For example, it has been observed that the lateral posterior fusiform gyrus responds more robustly to animate than inanimate objects, suggesting that there may be specific brain regions devoted to encoding visual properties of animate objects [11]. Other studies have shown bilateral activation of inferior occipital-temporal cortex for natural objects, as opposed to a left hemispheric activation for artifacts [12-14].

In addition to the literature on perceptual processing, neuroimaging and ERP studies on language processing provide evidence of category-specific functional dissociations. For example, left frontal anterior regions are active during processing of function words [15], temporal-parietal areas are active during processing of nouns [16], and abstract words were more likely than concrete words to activate anterior brain areas [17]. Verbs are more likely than nouns to activate anterior brain regions, including motor and premotor areas [18] and other left frontal areas [19], thus supporting the hypothesis.

According to Caramazza and coworkers [20,21], who proposed the *domain-specific *model of representation of conceptual knowledge, neural circuits dedicated to processing particular semantic categories are neuroanatomically and functionally segregated. These category-specific semantic systems may have originally evolved because rapid and efficient identification of objects from particular categories had survival advantages, and reflect an innate categorical organization of the brain. The model proposes that domain-specific representation systems, which are accessed by verbal and non-verbal stimuli, store conceptual information related to one particular category. According to this view, damage to one semantic system impairs all semantic information pertaining to one category (visual, functional, etc.). On the other hand, Tyler and Moss [22] proposed a model in which semantic categories (i.e., tools, vegetables) and domain (animate, inanimate) are not explicitly represented but are emergent properties of the structure and content of semantic representation, and categorical dissociations would depend on the specific patterns of brain activation in response to stimuli characterized by specific perceptual and functional features. For instance, distinctive or shared features of stimuli determine the degree of segregation or overlap in the locus of brain activation. Therefore, the sites of activation in response to "lion" and "canary" would be similar not because the words belong to the same domain (animate objects), but because they share domain-relevant perceptual properties (legs, eyes, faces, etc.). The authors are very critical about the theory postulating a clear neuroanatomical segregation of neural circuits devoted to processing of distinct semantic categories, and base their claim on the many inconsistencies present in the neuropsychological and neuroimaging literature failing to provide clear dissociations, especially for processing of living Vs. non-living things. Therefore, according to these and other authors [22-24] conceptual knowledge would be represented within a unitary distributed system without clear functional and neuroanatomical boundaries.

In this regard, Gerlach et al. [25] performed a positron emission tomography (PET) study in which they compared regional cerebral blood flow (rCBF) in two different conditions. The first condition was a categorization task in which subjects decided whether pictures represented artifacts or natural objects, and the other was a decision task in which subjects decided whether pictures represented real objects or non-objects. The categorization task was associated with activation in the left inferior temporal gyrus. The object decision task was associated with bilateral activation of the fusiform gyrus. The results suggest that the fusiform gyrus may be involved in first-pass processing of structural object properties, and the left inferior temporal gyrus may be involved in the analysis of functional and semantic object properties. However artifacts and natural objects often caused activation in the same regions within tasks. Therefore, the authors concluded that categorization and object recognition are not totally segregated processes. Similarly, results of other studies support the hypothesis of a distributed representation of visual objects in the ventral temporal cortex [23].

In summary there isn't a general agreement on the way conceptual knowledge is represented in our brain. As for the time course of neural processing related to object categorization several electrophysiological studies have examined the timing of brain evoked responses to determine when semantic knowledge is segregated in the human brain [8,26-30]. Ji and colleagues [28] found a specific involvement of right frontal cortex (145–200 ms) during categorization of vegetable/fruits which was absent in the topographic distribuition of animal-elicited ERPs, in a match/non-match S1–S2 paradigm. However a difference in familiarity or perceptual complexity of vegetable with respect to the animal exemplars (possibly accounting for the frontal involvement) was not considered in this study. The authors also found a greater posterior N1 to animals than vegetable/fruits. On the contrary, Sitnikova and colleagues [30] recorded ERPs in response to pictures of animal and tools and found an increase in negative potentials associated with tools compared to animals at left posterior and anterior areas in response to animals 200–500 ms after stimulus onset. They did not find an early (80–200 ms) effect of stimulus category in response to animate and inanimate objects. Similarly, Sim and Kiefer [29] presented 160 pairs of names of common objects, half of which were artifacts (including tools, furniture, means of transportation, and musical instruments) and half of which were natural (including animals, plants, fruits, and vegetables) in a feature verification task with verbal stimuli. Word pairs were formed such that objects were visually and/or functionally similar. In the first phase, the task was to decide whether the two objects belonged to the same category (natural or artifactual). ERPs showed that natural objects elicited a larger positive potential than artifacts over occipital-parietal areas in the N400 and late positive (LP) time windows, particularly over the right hemisphere. In the second phase, half of the subjects had to decide whether objects had similar shapes (visual judgment). The remaining subjects had to decide whether the function of objects was similar or dissimilar (functional judgment). ERPs were more positive to words that represented natural objects compared to those representing artifacts over the right occipito-parietal area, but only during the visual judgment task. The results suggest that the processing of natural categories depend relatively stronger on the activation of posterior brain areas, thus supporting the notion of a modality specific semantic system.

In a previous ERP study, Kiefer [8] investigated category-specific categorization mechanisms by comparing visual and verbal modalities with pictures and words referring to natural and artifactual categories. First a category probe was shown which was followed by a target stimulus. The task was to decide whether the target object was a member of the previously presented category. For example. probe = "animal", target = "cat". In the perceptual condition (i.e., Experiment 2) primers and targets were images of animals and tools. In the verbal condition, targets were written names of objects. Interestingly, there was an early inferior temporal N1 response that was greater to natural objects than artifacts, but only in response to images, not written words. The authors concluded that 1) there was greater activation in response to natural objects due to simultaneous activation of multiple competing exemplars, 2) perceptual information (i.e., images) was more relevant than linguistic information (i.e., written words) for natural entities, and 3) the greater relevance of perceptual information for natural objects was due to high within-category similarities.

In order to further investigate the neural mechanisms that support processing of objects belonging to different domains we used a perceptual categorization task without involving linguistic functions. At this purpose no verbal stimuli were used but only drawings of man-made objects and animals. ERPs to pairs of objects and animals (as well as mixed stimuli) were recorded while participants performed a super-ordinate categorization task. Different stimuli to be compared by viewers were not similar in shape. In order to be correctly recognized as belonging to the one or the other category their perceptual or functional properties were to be accessed as quickly and accurate as possible to perform a speeded response. The goal of the study was to investigate 1) if any difference in brain activation was observed as a function of stimulus semantic category (as suggested by previous literature), 2) more importantly, when exactly in time this difference was observable. In this regard, the event-related potentials technique is a very powerful tool for investigating the time course of information processing because of its capacity to record brain activity generated in different cortical and sub-cortical regions with a very high temporal resolution (1–2 ms).

While the majority of studies on the representation of conceptual knowledge in the brain involves linguistic coding, our intent was to investigate the emergence of semantic categorization in visual processing by means of a purely perceptual task. We predicted that if the observed differences consisted, for instance, in a greater response of visual areas for animals and of anterior and motor areas for man-made usable tools, within the first 150 ms of processing, this would suggest an early semantic catogorization emerging from the specific patterns of brain activation, and thus supporting a *modality-specific *representation of conceptual knowledge.

In this study subjects were asked to make a categorization judgement based on the semantic distinction between animate and inanimate stimuli based on image-specific structural and functional information. The task consisted in paying attention and responding to homogeneous pairs of animals (or artifacts) presented in a given field, while ignoring mixed configurations or location irrelevant stimuli. In this paper we report patterns of brain activity related to processing of animals vs. artifacts but independent of attentional factor (being a target or a non-target).

## Results and Discussion

### Behavioral data

An analysis of variance (ANOVA) demonstrated a significant effect of category on response latency (F[1,17] = 61.669, p < 0.001). Subjects responded much faster to animals (517 ms) than objects (564 ms). Neither visual field nor response hand affected reaction time (RT). An analysis of false alarms showed a significant effect of category (F[1,17] = 19.168, p < 0.001), with a higher percentage of errors made for artifacts (17.68) compared to animals (14.94).

### Electrophysiological data

The latency and amplitude of the early sensory C1 component (60–80 ms) and the P1 component (80–120 ms) were not affected by stimulus semantic category, indicating that stimuli were balanced in terms of luminance and spatial frequency (see Table 1 for a list of all statistical significances).

**Figure 1:**
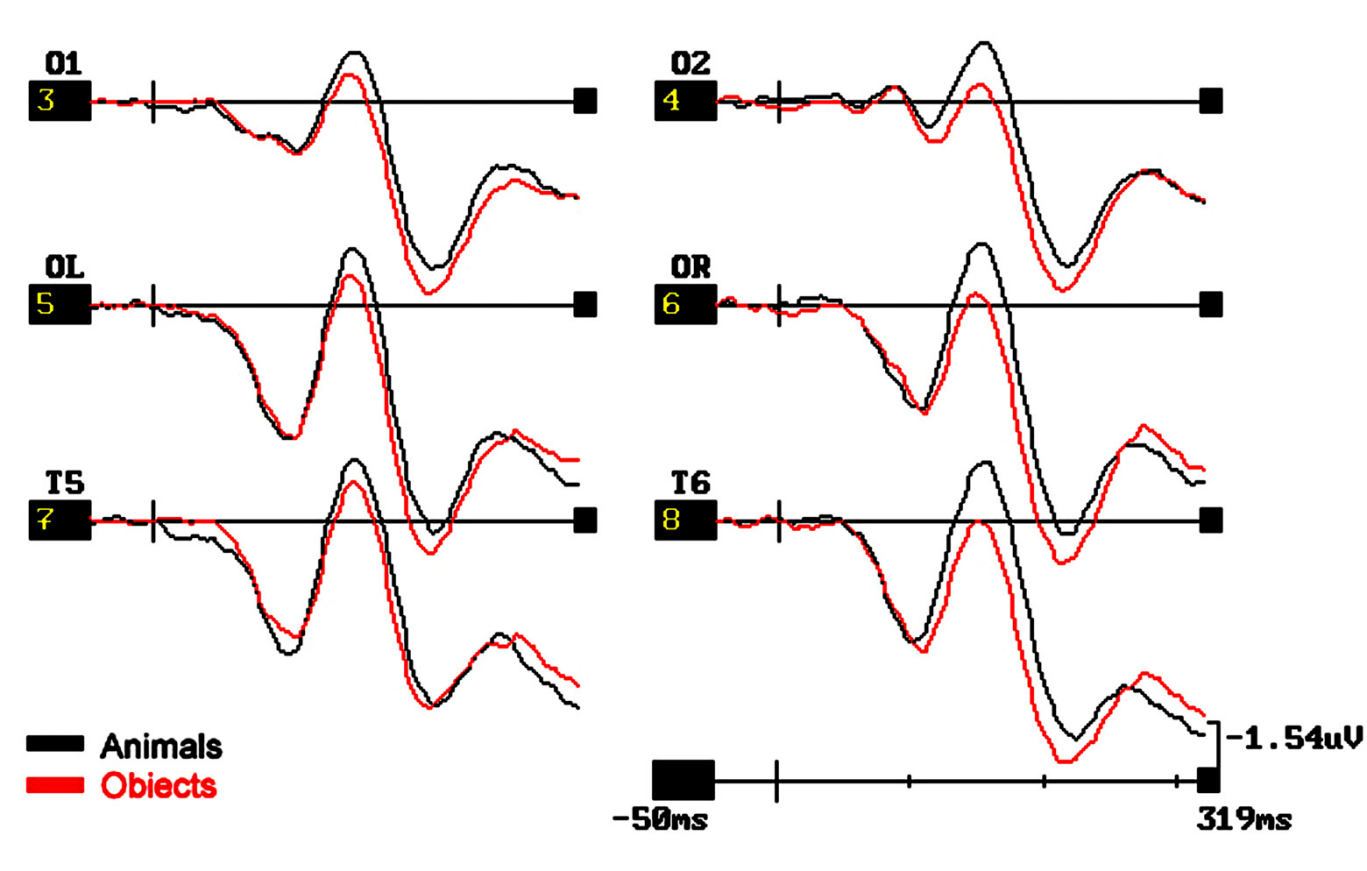
**ERP responses to animals and objects**. Grand average ERPs recorded at occipital, lateral occipital, and posterior temporal sites in response to animals and artifactual objects.

The analysis of N150 peak amplitude revealed an effect of stimulus category with larger N1 responses to animals (-2.9 μV) than artifacts (-1.71 μV). Post-hoc analysis of the significant category × hemisphere interaction suggested that, at this latency, the right hemisphere was significantly more sensitive than the left to stimulus category, with a greater difference in the response to animals than objects (right hemisphere, animals = -3.04 μV, artifacts = -1.51 μV, difference = 1.47 μV; Tukey test p < 0.001; left hemisphere, animals = -2.76 μV, artifacts = -1.92, difference = 0.84 μV; Tukey test p < 0.012), as shown in Fig. 1.

**Figure 2:**
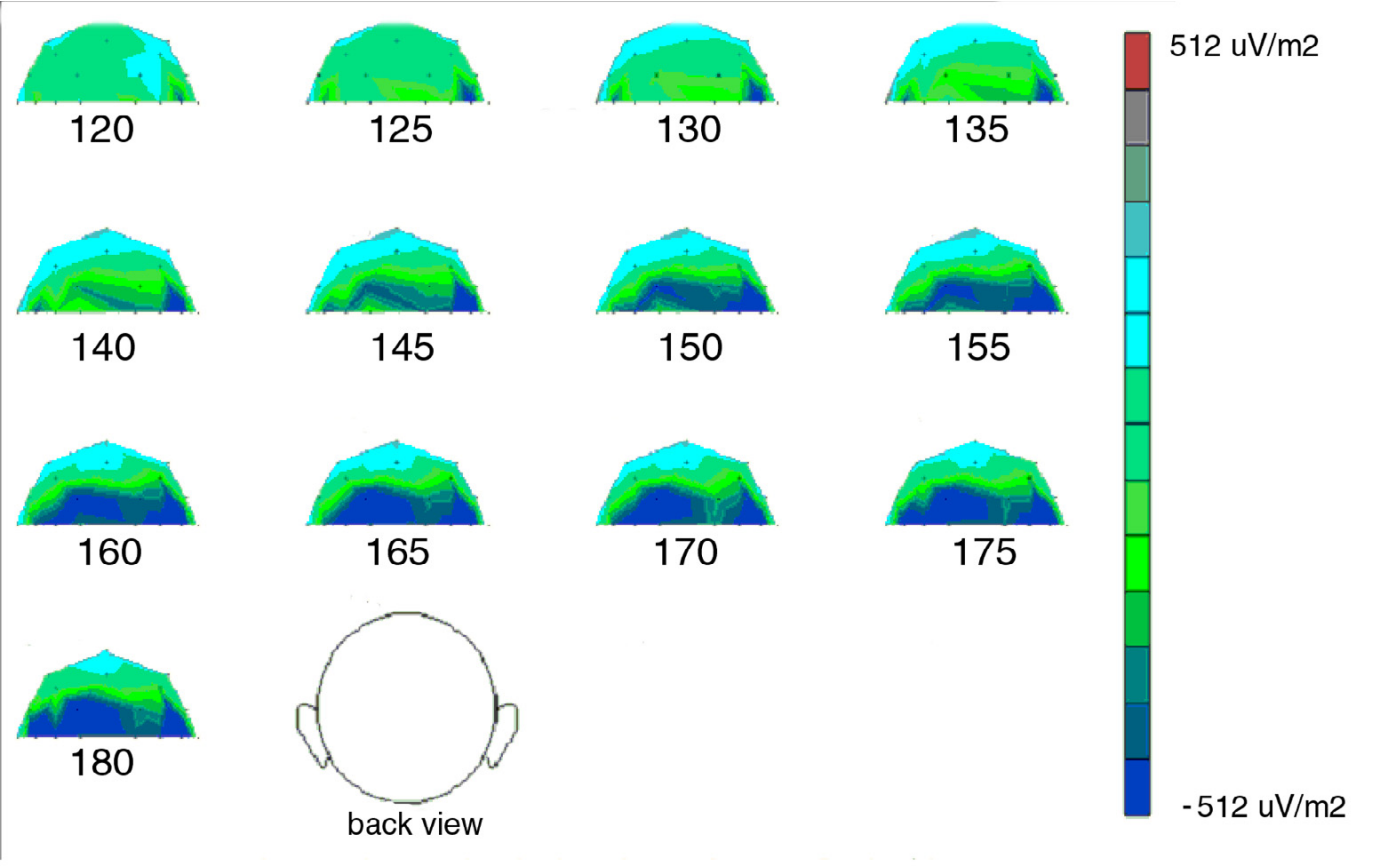
**SCD difference maps for N1 (animals-objects)**. Temporal series of scalp current density difference maps obtained by subtracting brain activity related to man-made objects from activity related to animals every 5 ms during the 120–180 ms post-stimulus interval.

Temporal series of scalp current density (SCD) difference maps were computed between 120–180 ms with a pass of 5 ms. Differences were obtained by subtracting SCD maps for artifacts from those for animals. The earlier involvement of right posterior temporal cortex in the discrimination between the two shape categories is evident in Fig. 2.

An ANOVA of the N270 latency showed a significant interaction of category × visual field × electrode. Post-hoc comparisons indicated that the N2 component had a shorter latency at lateral occipital and posterior temporal sites in response to animals (267 ms) compared to artifacts (273 ms) that were presented in the left visual field (p < 0.04).

The amplitude of the N2 component, larger at mesial occipital sites, was affected by stimulus category, in interaction with hemisphere. Again, the right hemisphere was more sensitive to semantic category: images of objects that were presented in the left visual field elicited a larger negative (2.23 μV) potential than images of animals (3.33 μV) over the right hemisphere, as demonstrated by a significant category × visual field × hemisphere interaction and post-hoc analysis (p < 0.01).

**Figure 3:**
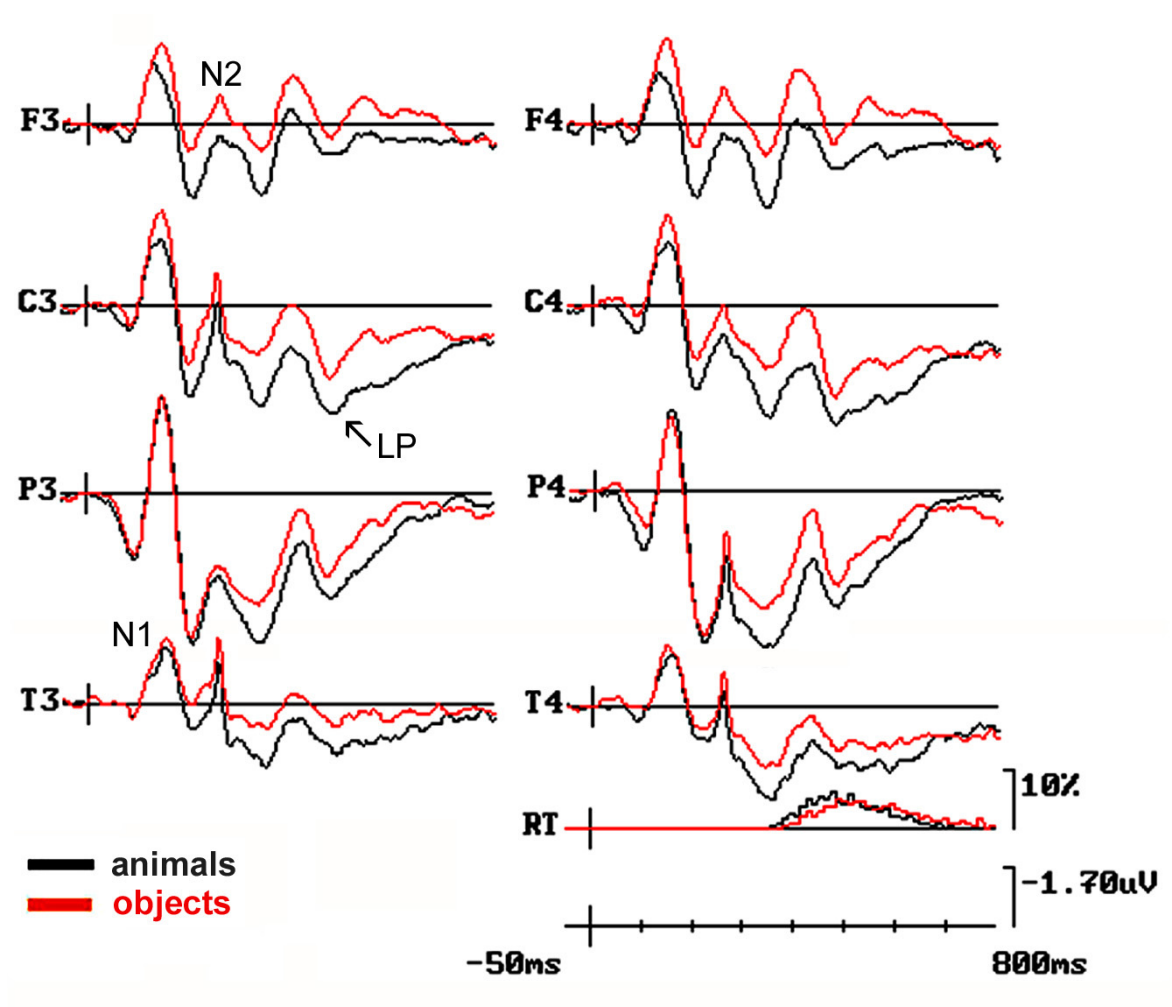
Grand average ERPs recorded at temporal, parietal, central and frontal sites in response to stimuli of the two categories (animals and man-made objects).

The anterior N1 (130–160 ms) larger at central sites, showed a similar amplitude in response to contralateral and ipsilateral stimuli in the RH and a much larger response to contralateral stimuli at left sites, where it reached its maximum amplitude, as shown by hemisphere × visual field interaction. It was strongly affected by stimulus category, being much larger in response to artifacts (-2.13 μV) than to animals (-1.56 μV), as shown in Fig 3. This effect showed an anatomic specificity (category × electrode interaction) with a significant animal/object difference at anterior (frontal/central) but not than temporal sites, as shown by post-hoc comparisons (Temp: A = -1.3, O = -1.62; Centr: A = -1.92, O = -2.54; Front: A = -1.45, O = -2.21 μV).

**Figure 4:**
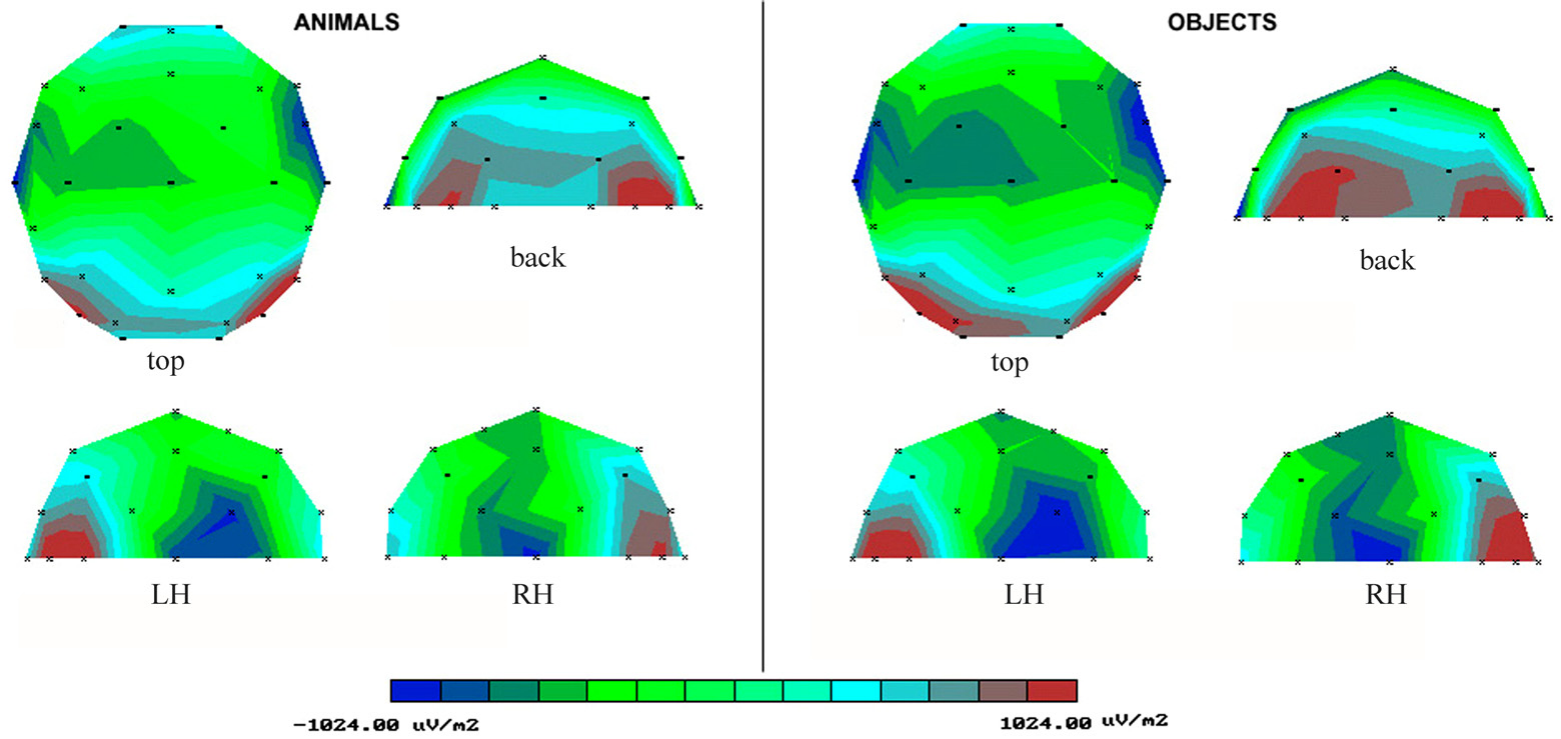
**SCD difference maps for N2 (animals vs. objects)**. Top, back, and lateral views of SCD maps computed at N2 peak latency (250 ms after stimulus onset) according to stimulus category.

Anteriorly, the N2 component (200–260) was larger at temporal sites and exhibited a marked left hemispheric asymmetry as also visible in maps of Fig. 4. Overall N2 was sensitive to stimulus category being larger in response to artifacts (0.15 μV) than to animals (1.01 μV). The category dissociation was bilateral at frontal/central sites, and strongly lateralized toward the left hemisphere at temporal sites. Indeed N2 showed its maximum amplitude in response to artifacts (-0.64 μV) than animals (0.22 μV) at left temporal site, as shown by significant category × electrode and category × electrode × hemisphere interactions (see Table 1)

The ANOVA performed on latency values of parietal P3, which was earlier in the left than right hemisphere, demonstrated a significant category × hemisphere interaction, with earlier P3 peaks in response to artifacts (345 ms) compared to animals (335 ms) at left sites (post-hoc Tukey test, p = 0.003).

**Figure 5:**
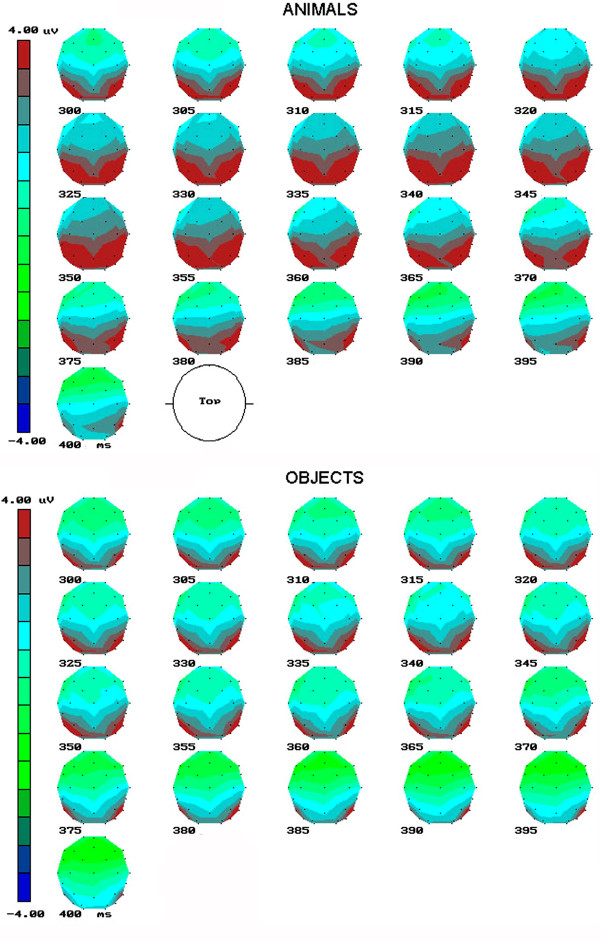
Time series of topographic maps computed in the P300 latency range (300–400 ms) with a pass of 5 ms as a function of stimulus category. Top: Responses to animals; bottom: Responses to man-made objects; top view.

**Figure 6:**
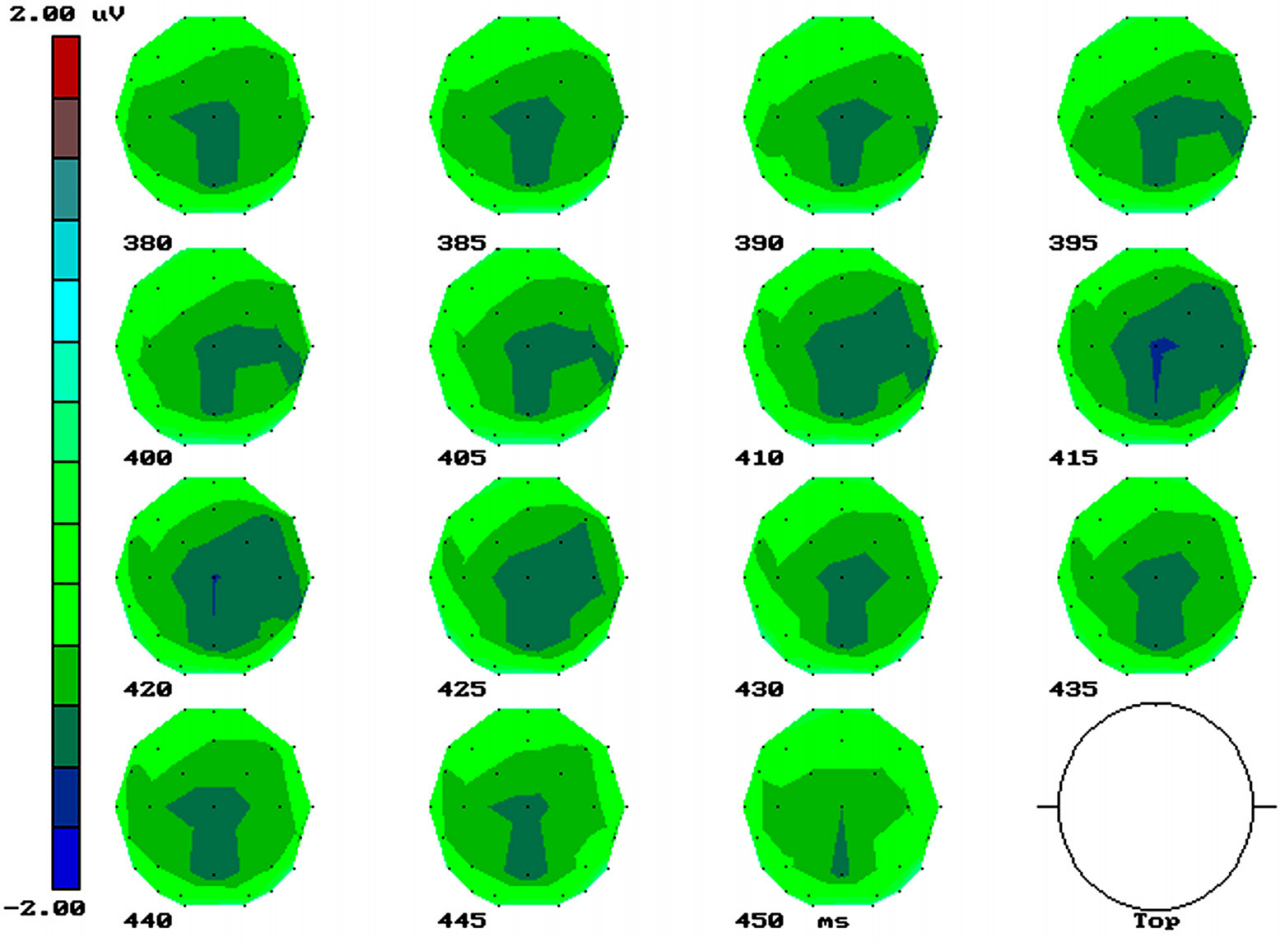
Time series of difference maps in the N4 latency range (380–450 ms) computed by subtracting the scalp voltage distribution of ERPs in response to animals from those in response to objects. The maps show a larger activation in right central-parietal areas during categorization of man-made objects.

P300, which reached its maximum amplitude at parietal sites, was strongly affected by stimulus being larger to animals than artifacts (Fig. 5). In contrast to the P3 component, the N4 response at central-parietal sites was greater to artifacts (0.59 μV) than animals (1.93 μV), as shown by a significant effect of category. Fig. 6 shows a time series of difference maps computed in the N4 latency range (380–450 ms) that were obtained by subtracting the scalp voltage distribution of ERPs to animal stimuli from those to artifact stimuli and showing the distribution of negative potentials that were larger in response to artifacts. In the following interval (450–520 ms), the LP component, showing a midline distribution, was very sensitive to stimulus category, being it larger in response to animals (4.09 μV) than artifacts (3.20 μV).

In this study subjects were required to press a button in response to target pairs of animals or artifacts without directly accessing or providing verbal or conceptual representation of the items. Their complexity and familiarity was rated post-hoc according to the instructions of Snodgrass and Vanderwart [31]. Our results demonstrated that stimuli for both categories were at an identical level of complexity (i.e., neither complex nor simple, see Table 2), with a slightly greater level of familiarity for artifacts (that tended to be judged as fairly familiar) than for animals (that tended to be judged as neither familiar nor unfamiliar). Response latencies were not affected by stimulus complexity or familiarity. RTs were faster to equally complex but less familiar objects (i.e., animals).

### Early perceptual effects

Our data showed an early effect (~150 ms post stimulus) in right posterior visual areas. Indeed, the right occipital-temporal cortex was significantly more sensitive than the left to stimulus category in the 120–300 ms interval. A similar increase in the early negative response to natural but not artifactual stimuli was observed by Kiefer ([8] Exp. 2 perceptual condition) who reported larger N170 potentials over inferior temporal-occipital regions in response to images of animals compared to images of tools. The topography of the category-related ERP effect is also consistent with results of neuroimaging studies, which suggest that processing natural stimuli is more dependent on the visual association cortex in occipital-temporal areas than is processing artifactual stimuli [13,29,32,33]. There is also evidence that the right occipital-temporal cortex, and to a lesser extent its contralateral counterpart, is involved in the representation of natural categories (e.g., [4,8]). In addition, the greater activation in right visual cortex in response to animals compared to artifacts may be related to specific activation of the visual face area, a region in the central occipital-temporal cortex that includes the fusiform gyrus (fusiform face area, FFA) and has been shown to respond more robustly to faces than to other objects, such as houses, tools, and inverted faces. Activation of this area has been identified in the posterior N170 component [34,35].

On the other hand, the larger frontal/central N1 (130–160) (and subsequent anterior temporal N2) in response to artifacts than animals is consistent with the data by Antal and colleagues [26,27] who found a greater N1 to non-animals (natural and urban scenes, objects, flowers, fruits) than animals at frontal sites (F3, F4, Fz, Cz) in a visual categorization task.

It is also consistent with the literature supporting the notion of an anterior brain activation for accessing knowledge related to actions and motoric patterns such as verbs as opposed to nouns [18,19], manipulable tools as opposed to animals [36,37] or manipulable objects in natural as opposed to awkward grips [38].

The differences we observed in early brain activation during stimulus processing and recognition are likely due to structural differences in animals and artifacts that might have a strong effect on sensory and perceptual processing. Animals are more homomorphic (i.e., they all have heads and eyes) than artifacts [4]. Conversely, inanimate objects share fewer characteristics and may belong to an infinite set of specimens. Therefore, object processing appears to be performed by a set of multi-processes that analyze physical and functional properties of an object. On the other hand, recognizing animals might involve only sensory identification of physical features [39] such as faces, eyes, and legs. Interestingly, it was suggested that processing stimuli belonging to a specific semantic category reflects processing demands that are jointly determined by representational structure and the particular task being performed [40].

### Later ERP effects

Processing images of animals was associated with faster RTs, larger occipital-temporal N1 components and larger parietal P3 and LP components. These results suggest that animal recognition was less demanding than object recognition. The much larger parieto/occipital activation related to animal than artifacts at P3 level (300–400 ms) displayed in maps of Fig. 5, might be interpreted also as greater involvement of visual sensory areas in the processing of associate semantic features. On the other hand, processing images of artifacts was associated with slower RTs and larger posterior N2, frontal/central N1, anterior temporal N2 and centro-parietal N400 responses. These effects were probably linked to the larger positive potentials elicited by animal images. They are consistent with those described by Kiefer [8] for the perceptual study (Experiment 2), showing shorter RTs and smaller central-parietal N400 responses to natural compared to artifactual stimuli (in the incongruent condition). On the other hand, they do not agree with the findings of Sitnikova et al. [30], who reported larger negative components 200–600 ms after presentation of animals compared to tools and no differences in RTs or accuracy for stimuli from different categories. The authors interpreted their results in terms of quick, direct access to functional semantic representation for tools compared to animals.

One might hypothesize that the larger N400 response to artifacts compared to animals is related to differential inter-category stimulus familiarity. Indeed, the right central-parietal linguistic N400 component, which shared topographic distribution and functional properties with the image-evoked N400 in the present study, is described in the literature as having greater amplitude to low compared to high frequency items and to pseudo-words compared to words. That is, the N400 potential is larger in response to unfamiliar compared to familiar items [41,42]. Kiefer [8] reported a smaller negative potential over the right parietal region in response to natural objects compared to artifacts, with stimuli that had been rated as equally typical but not equally familiar or complex. In that study, stimulus complexity and familiarity was rated post-hoc according to the instructions of Snodgrass and Vanderwart [31]. Results showed that artifacts were rated as slightly more complex (2.8 vs. 2.5) and less familiar (2.8 vs. 3.1) than animals. In that case, the N400 effect might have hardly been interpreted as an index of increased response to less familiar pictures. Furthermore, in our study, animals and artifacts were judged as equally complex in perceptual structure, and artifacts were judged as only slightly more familiar than animals (3.6 vs. 3.3). Therefore, late category effects on the N400, which in the Kiefer's study [8] were observed independent of input modality (pictures, words), cannot be accounted for by factors like visual complexity or familiarity and according to Kiefer might reflect modality-specific feature-representation within the semantic system. In other words, it might reflect a stronger access to visual semantic features stored in posterior brain regions for animals than artifacts.

The N400 component is believed to index semantic integration processes and the difficulty with which new information is processed and integrated with previously stored information, including semantic and world knowledge [43]. Accordingly, a larger N400 in the right central-parietal area that is clearly visible in Fig. 6 might also reflect a difference in semantic categorization processes. Indeed, our behavioral data, showing slower RTs and more frequent categorization errors for artifacts compared to animals, support this interpretation. Decision-making processes are likely to be more difficult for less homogeneous items than items that share more perceptual (i.e., heads, eyes, legs) and semantic (i.e., animate objects that move, eat, breath, and make noise) properties. And indeed Recently, Paz-Caballero and colleagues [44] provided evidence of a dissociation between difference in semantic domains for natural vs. artifactual entities, and difference in task difficulty. In their study categorization of natural stimuli was associated with faster RTs and larger late positivity (exactly like in the present study), and was interpreted with the notion that natural stimuli are easier to correctly categorize than artifactual stimuli, due to greater perceptual similarity, and as supported by available literature [8,45]. Artifactual objects may be even slightly more familiar than objects if presented singularly (as in the present study) but difficult to discriminate if presented randomly mixed.

### General Discussion

Our data provide evidence of differential brain activation during visual categorization of items belonging to animal and artifact categories. At early latency stages (N170) the right occipital-temporal cortex exhibited larger potentials to familiar, homomorphic, visually salient objects with faces (i.e., animals) compared to artifacts. On the other hand, RTs were slower, P300 was smaller, frontal/central N1 and anterior temporal N2 and N400 potentials were larger to equally complex and familiar artifacts, especially usable man-made objects. These data are consistent with literature that supports the idea that activation of anterior brain regions is greater during perception of manipulable tools compared to non-usable objects [36,37]. For example, a recent fMRI study [37] demonstrated that viewing graspable tools, but not shapes, activated motor-related regions of cortex (posterior middle temporal gyrus, ventral premotor area, posterior parietal cortex). The authors concluded that the functional identity of graspable objects influences the extent to which they are associated with motor representations.

## Conclusion

Overall, our data are compatible with a model in which sensory and action-related semantic features of objects are represented in modality-specific brain areas. According to this model, category-specific differences in the type and distribution of neural response depend on several factors, including object imageability; motor association [18,19,36-38]; abstractness [17]; content of sensory information [39]; complexity; familiarity (e.g., [46] for humans, [47] for monkeys); the presence of learning-independent cues, such as eyes, faces, and hands (e.g., [34,35] for faces); learning (e.g., [32,48] for language reading); homomorphism [4]; content of acoustic or phonetic information [49]; content of spatial information (e.g., [50] for places); complexity of spatio-temporal mnestic coordinates (e.g., [51] for proper and common name categories); and not necessarily semantic domain.

However, the present data are not inconsistent with the view that there may be some overlapping in the way objects belonging to different semantic domains are represented in the brain, in line with other neuroimaging data. For example, Haxby and colleagues [52] showed that, while it is possible to identify specific regions of ventral temporal cortex responding maximally to a specific object category (e.g., man-made objects or cats), patterns of response that discriminate among all categories are found even within cortical regions that respond preferentially to one category.

## Methods

### Subjects

Eighteen right-handed healthy undergraduates (7 males, 11 females, mean age = 23.5 years) participated in the study as unpaid volunteers. All participants had normal or corrected-to-normal vision. The study was approved by the ethics committee of the University of Milano-Bicocca and was conducted in accordance with ethical standards (Helsinki, 1964). Informed written consent was obtained from each participant.

### Stimuli and Procedure

**Figure 7:**
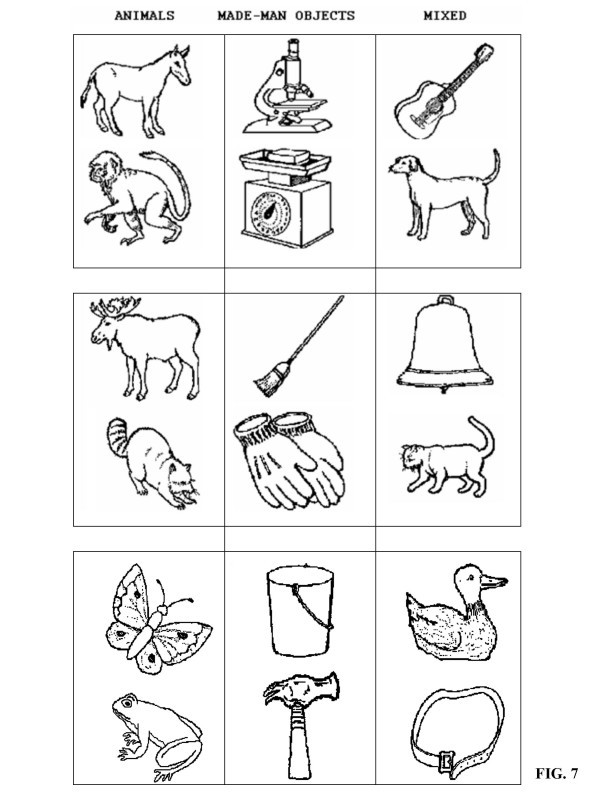
Examples of black and white drawings used as stimuli for animal pairs, object pairs, and mixed pairs.

Stimuli consisted of 672 pairs of vertically arranged images. Table 2 provides a list of all objects represented by the stimuli. Familiarity and perceptual complexity of all images were evaluated post-hoc by an independent sample of 20 subjects, as described in the legend . Stimuli were black pencil drawings on a white background. They could belong to three different semantic categories: i) 168 homogeneous pairs of animals, ii) 168 homogeneous pairs of artifacts, and iii) 336 mixed pairs (animals and artifacts). Fig. 7 shows 3 exemplars of stimuli for the various categories. Homogeneous and mixed stimuli pairs were presented with equal probability. They were created by randomly mixing 44 × 2 exemplars of animals and artifacts that are listed in Table 2.

Stimuli subtended 6° 22' 12" of visual angle in height and 3° 49' 12" in width. All stimuli were equiluminant (17.96 candela/m^2^). Stimulus pairs were randomly presented for 250 ms in the right or left visual hemifields, 2° 30' of eccentricity along the horizontal meridian. The interstimulus interval varied from 900 to 1200 ms.

Participants sat in an acoustically and electrically shielded cabin ~135 cm from a PC monitor. They were instructed to fixate on a small cross located at the centre of the screen and avoid any body or eye movements. The task consisted in deciding whether stimulus pairs belonged to the same category (animals or artifacts, depending on task requirements) within a given visual field (LVF or RVF). Therefore it was a conjoined space- and object-based visual selective attention task. The category to be responded to and the relevant visual field were announced by the experimenter at the beginning of each run. Participants had to press a button to targets as accurately and quickly as possible with the index finger of the left or right hand. Mixed pairs and non-target stimuli were to be ignored. Task conditions and responding hands were balanced and randomized across subjects.

### EEG recording and analyses

Electroencephalograms (EEGs) were continuously recorded from 30 scalp electrodes mounted in an elastic cap. The electrodes were located at frontal (Fp1, Fp2, FZ, F3, F4, F7, F8), central (CZ, C3, C4), temporal (T3, T4), posterior temporal (T5, T6), parietal (PZ, P3, P4), and occipital (O1, O2) scalp sites of the International 10–20 System. Additional electrodes were placed at an anterior frontal site (AFz), halfway between frontal and central sites (FC1, FC2, FC5, FC6), central and parietal sites (CP5, CP6), parietal and occipital sites (PO3, PO4), and posterior temporal and occipital sites (OL, OR). Vertical eye movements were recorded by two electrodes placed below and above the right eye, and horizontal eye movements were recorded by electrodes placed at the outer canthi of the eyes. Linked ears served as the reference lead. The EEG and electrooculograms (EOGs) were amplified with a half-amplitude band pass of 0.02–50 Hz. Electrode impedance was kept below 5 kΩ. Continuous EEGs and EOGs were digitized at a rate of 512 samples/sec. EEG epochs were synchronized with the onset of stimuli presentation.

Computerized rejection of electrical artifacts was performed before averaging to discard epochs in which eye movements, blinks, excessive muscle potentials, or amplifier blocking occurred. The artifact rejection criterion was a peak-to-peak amplitude exceeding 50 μV, and the rejection rate was ~5%. ERPs were averaged off line from 100 ms before stimulus onset to 850 ms after stimulus onset. For each subject, distinct ERP averages were obtained according to stimulus category. ERP components were identified and measured with reference to the baseline voltage averages over the interval from -100 ms to 0 ms relative to stimulus onset.

Mean area values of C1 and peak latency and amplitude values of P1 components were measured at occipital (O1, O2), lateral occipital, (OL, OR), posterior temporal (T5, T6), and occipital-parietal (PO3, PO4) electrode sites 60–80 ms after stimulus onset and 80–120 ms after stimulus onset. Peak latency and amplitude values of N1 and N2 components were recorded at occipital (O1, O2), lateral occipital (OL, OR) and posterior temporal (T5, T6) sites 120–180 ms and 240–300 ms after stimulus onset. Mean area values of anterior N1 and N2 components were measured at temporal (T3 and T4), central (C3 and C4) and frontal (F3 and F4) sites in between 130–160 and 200–260 ms, respectively. Peak latency and amplitude values of P300 and mean area values of LP and N400 were measured at parietal (P3, Pz, P4) and central (C3, Cz, C4) sites. P300 peak was identified and measured in the latency range of 300–400 ms. N400 and LP were identified and measured in the time windows of 380–450 and 450–520 ms, respectively.

### Statistical analyses

In the statistical analysis, any RTs under 140 ms, or exceeding the mean ± 2 standard deviations, were excluded. The RT data, including arc sin transformed percentage of errors and latency and amplitude of the major ERP components, were subjected to multifactorial repeated-measures ANOVA. Three-way repeated measures ANOVA were performed on behavioral responses. Factors included in the analyses were visual field (left, right), response hand (left, right), and stimulus category (animal, artifact). Four-way repeated-measures ANOVA were performed on electrophysiological responses. Factors included in the analyses were visual field, stimulus category, electrode" (depending on ERP component), and hemisphere (left, right). Post-hoc Tukey tests were used to for multiple comparisons of mean values. Multiple comparisons of means were done by post-hoc Tukey tests.

McCarthy-Wood correction sometimes used to normalize ERP amplitudes was not applied to our data, in line with recent findings in the literature [53].

Topographical voltage maps of ERPs were made by plotting color-coded isopotentials derived by interpolating voltage values between scalp electrodes at specific latencies. SCDs (second spatial derivative of the potential) were computed from the spherical splineinterpolation of the surface voltage recordings made from all 30 channels.

## Authors' contributions

AMP conceived of the study, coordinated data acquisition and analysis, interpreted the data, and drafted the manuscript. MDZ participated in the design of the study, collected data, and performed statistical analyses. AZ participated in the study design and coordinated data acquisition. All authors read and approved the final manuscript.

## Figures and Tables

**Table 1:** Table of statistical significances obtained for various ERP components of interest.

**ANOVA significances**			
**Factors**	F	**df**	p
**C1 amplitude**			
VF × H	12.82	1,17	<0.0025
**P1 latency**			
VF × H	52,35	1,17	<0.001
E	3,693	3,51	<0.02
**N1 latency**			
VF × H	29.17	1,17	<0.001
**N1 amplitude**			
Category	41.43	1,17	<0.001
Cat × H	21.32	1,17	<0.001
**Occip N2 latency**			
Cat × VF × E	3.46	2,34	<0.043
**Occip. N2 amplitude**			
E	24.30	2,34	<0.001
VF × H	9.15	1,17	<0.008
VF × H × E	3.79	2,34	<0.03
Cat × VF × H	8.15	1,17	<0.01
**Anterior N1 (130–160)**			
E	4.4	3.24	<0.02
Cat	6.92	1,17	<0.02
Cat × E	3.41	3,24	<0.05
Cat × H	21.15	1,17	<0.001
Cat × E × E	6.35	2,34	<0.005
**Anterior N2 (200–260)**			
E	5.6	2,34	<0.008
E × H	3.97	2,34	<0.03
VF × E × H	8.79	2,34	<0.001
Cat	12.03	1,17	<0.003
Cat × E	5.38	2,34	<0.009
Cat × E × H	4.01	2,34	<0.03
**P3 latency**			
VF × H	17.45	2,34	<0.001
VF × H × E	5.59	2,34	<0.008
H	4.36	2,34	<0.02
Cat × H	6.57	2,34	<0.004
**P3 amplitude**			
E	8.78	1,17	<0.009
VF × E × H	6.20	2,34	<0.005
Cat	15.58	1,17	<0.001
**N400 area (380–450)**			
VF × H	62.6	2,34	<0.001
Cat	37.94	1,17	<0.001
**LP area (450–520)**			
E	3.11	2,34	= 0.057
VF	4.62	1,17	<0.05
Cat	6.95	1,17	<0.02

**Table 2:** List of all animals and artifacts used to compose 672 pairs of stimuli.

**ARTIFACTS**			**ANIMALS**		
	**Familiarity**	**Complexity**		**Familiarity**	**Complexity**
*Anchor*	3.0	2.55	*Baboon*	2.5	4.2
*Armchair*	4.05	2.65	*Bat*	3.6	4.55
*Bandaid*	4.15	1.3	*Bird*	3.4	3.65
*Banjo*	2.85	3.7	*Camel*	3.6	2.9
*Bathtub*	2.9	3.4	*Cat1*	4.0	3.8
*Bed*	3.95	2.75	*Cat2*	3.2	2.4
*Belt*	3.6	1.65	*Cow*	2.9	3.15
*Bike*	4.4	4.25	*Deer*	3.3	2.8
*Book*	3.95	1.8	*Dinosaur*	3.1	1.95
*Bottle*	2.35	1.15	*Dog1*	4.0	2.45
*Broom*	4.15	2.3	*Dog2*	3.75	2.35
*Brush*	3.2	3.3	*Dog3*	3.7	2.4
*Canoe*	3.0	3.8	*Dolphin*	3.63	1.35
*Car*	4.25	3.65	*Donkey*	3.2	2.25
*Cart*	2.95	2.9	*Duck1*	3.6	3.05
*Chair*	4.2	2.35	*Duck2*	3.9	2.85
*Clock*	4.35	2.6	*Falcon*	3.35	4.3
*Comb*	4.4	2.75	*Fawn*	3.4	3.5
*Compass*	2.4	3.5	*Fox*	2.45	3.6
*Crib*	3.3	3.1	*Goat*	3.0	2.4
*Doll*	3.25	3.85	*Gorilla*	2.9	4.3
*Door*	4.2	3.05	*Hen*	3.25	3.7
*Fork*	4.05	2.95	*Horse*	3.7	3.1
*Garbage*	3.6	2.4	*Kangaroo*	3.2	2.2
*Glass*	3.65	1.9	*Lamb*	3.3	3.55
*Globe*	3.9	3.2	*Leopard*	3.1	4.3
*Guitar*	4.0	3.45	*Lizard*	2.4	3.3
*Ladder*	3.95	2.2	*Llama*	2.65	2.95
*Mailbox*	3.4	2.9	*Lynx*	2.4	3.75
*Microscope*	3.95	3.9	*Monkey*	3.4	3.75
*Motorboat*	3.8	3.85	*Moose*	3.25	2.6
*Paint*	3.15	3.15	*Mouse*	3.95	2.75
*Piano*	4.05	4.7	*Octopus*	2.85	4.35
*Pipe*	3.4	2.7	*Panda*	3.55	3.3
*Purse*	3.5	3.6	*Parrot*	2.45	4.15
*Radio*	2.9	4.65	*Peacock*	2.6	4.8
*Scale*	3.35	3.7	*Penguin*	3.95	3.25
*Spoon*	3.35	2.9	*Pig*	3.5	1.25
*Telescope*	3.3	3.75	*Rabbit*	3.85	1.95
*Television*	4.0	3	*Raccoon*	2.55	3.3
*Trumpet*	3.25	3.7	*Seagull*	3.1	2.65
*Tuba*	3.4	3.5	*Sparrow*	3.45	3.55
*Typewriter*	3.6	4.45	*Spider*	3.6	3.6
*Vacuum cleaner*	4.3	3.85	*Tiger*	3.6	4.45
**Total**	**3.6208**	**3.1318**		**3.2761**	**3.1986**

Familiarity and complexity ratings [27]were obtained from an independent group of 20 subjects with 5-point scales. For the familiarity rating scale 5 = very familiar, 4 = fairly familiar, 3 = neither familiar nor unfamiliar, 2 = not very familiar, and 1 = unfamiliar. For the complexity rating scale 5 = very complex, 4 = fairly complex, 3 = neither complex nor simple, 2 = fairly simple, and 1 = very simple. There were no differences in complexity rating of animals (3.1986) and artifacts (3.1318). There was a small but significant difference in familiarity ratings of animals (3.2761) and artifacts (3.6208), as shown by a one-way analysis of variance (F1 [19] = 13.548; p < 0.001). Access to verbal or semantic properties of items was not required. Ten subjects rated the familiarity of all objects (randomly mixed) before rating their complexity, and 10 subjects rated the complexity of all objects before rating their familiarity.
